# Novel hypotheses emerging from GWAS in migraine?

**DOI:** 10.1186/s10194-018-0956-x

**Published:** 2019-01-11

**Authors:** Arn M. J. M. van den Maagdenberg, Dale R. Nyholt, Verneri Anttila

**Affiliations:** 1 Department of Human Genetics, Leiden University Medical Centre, Leiden, The Netherlands; 2 Department of Neurology, Leiden University Medical Centre, 9600, 2300 RC Leiden, The Netherlands; 3 School of Biomedical Sciences, Faculty of Health, Queensland University of Technology, Brisbane, QLD Australia; 4 Institute of Health and Biomedical Innovation, Queensland University of Technology, Brisbane, QLD Australia; 5 Analytical and Translational Genetics Unit, Department of Medicine, Massachusetts General Hospital and Harvard Medical School, Boston, MA USA; 6 Stanley Center for Psychiatric Research, Broad Institute of MIT and Harvard, Cambridge, MA USA; 7 Program in Medical and Population Genetics, Broad Institute of MIT and Harvard, Cambridge, MA USA

**Keywords:** Genetics, Genome-wide association study, Disease pathway, Single-nucleotide polymorphism

## Abstract

Recent technical advances in genetics made large-scale genome-wide association studies (GWAS) in migraine feasible and have identified over 40 common DNA sequence variants that affect risk for migraine types. Most of the variants, which are all single nucleotide polymorphisms (SNPs), show robust association with migraine as evidenced by the fact that the vast majority replicate in subsequent independent studies. However, despite thorough bioinformatic efforts aimed at linking the migraine risk SNPs with genes and their molecular pathways, there remains quite some discussion as to how successful this endeavour has been, and their current practical use for the diagnosis and treatment of migraine patients. Although existing genetic information seems to favour involvement of vascular mechanisms, but also neuronal and other mechanisms such as metal ion homeostasis and neuronal migration, the complexity of the underlying genetic pathophysiology presents challenges to advancing genetic knowledge to clinical use. A major issue is to what extent one can rely on bioinformatics to pinpoint the actual disease genes, and from this the linked pathways. In this Commentary, we will provide an overview of findings from GWAS in migraine, current hypotheses of the disease pathways that emerged from these findings, and some of the major drawbacks of the approaches used to identify the genes and pathways. We argue that more functional research is urgently needed to turn the hypotheses that emerge from GWAS in migraine to clinically useful information.

## Background

It has long been recognised that migraine is a disease with a strong genetic component [1–3]. Migraine runs in families, and epidemiological studies in twins and families have indicated that risk for migraine is conferred by a combination of genetic and environmental factors, both contributing equally [2, 3]. These studies also indicated that the genetic contribution seems stronger in migraine with aura than the more common migraine without aura subtype. Considerable progress has been made with elucidating the pathophysiological mechanisms in migraine. Evidence is accumulating that cortical spreading depolarisation (CSD) is the electrophysiological substrate of the migraine aura [4, 5]. Activation of the trigeminovascular system that consists of meningeal perivascular nerves, the trigeminal ganglion and brainstem centres reaches thalamus and ultimately the cortex to give the sensation of head pain in migraineurs during attacks [6]. Several animal studies showed that CSD can activate the headache mechanisms [7, 8], but proof that this also occurs in humans is essentially lacking. Knowledge on the underlying molecular mechanisms, to large extent, comes from genetic studies of very rare monogenic forms of migraine — i.e., hemiplegic migraine and syndromes in which migraine is prominent (for review see [9]). In brief, genes in familial hemiplegic migraine (FHM) (*CACNA1A*, *ATP1A2* and *SCN1A)* encode subunits of ion transporters (neuronal voltage-gated Ca_V_2.1 Ca^2+^, Na_V_1.1 Na^+^ channels, and glial Na^+^K^+^ ATPases, respectively) and functional studies in cellular and animal models suggest neuronal hyperexcitability as a common theme. Genes with vascular and/or glial cell function emerged from investigating syndromes in which migraine is prevalent, such as *NOTCH3* in cerebral autosomal dominant arteriopathy with subcortical infarcts and leukoencephalopathy (CADASIL) and *CSNK1D* in familial advanced sleep phase syndrome (FASPS). Since one such high impact mutation is causative for disease, the identification of these genetic factors directly benefits the clinical diagnosis of patients with these rare disorders and may lead to the development of better treatment.

Parallel research aimed to identify genetic factors for the common forms of migraine, foremost migraine with aura and migraine without aura, suggests that these migraine types are brought about by a combination of multiple genetic variants, each with low impact, and environmental factors [10]. The most effective approach thus far to identify genetic factors for these forms of migraine are genome-wide association studies (GWAS), which test for differences in allele frequencies of several million single nucleotide polymorphisms (SNPs) spread over the genome in large groups of cases and controls [11]. Allelic differences at SNPs with a *p*-value < 5 × 10^− 8^ are taken as proof that a migraine risk factor is located at that position. Due to their small effect size (allelic odds ratio of 1.03–1.28), no single SNP can have clinical use in migraine risk prediction; however, one can envisage that combined knowledge from many variants will highlight which genes and pathways are involved in migraine pathophysiology, as well as more direct applications through approaches like polygenic risk scoring, where additive effects of multiple migraine risk SNPs can be used to score patients and then analyse those scores against clinical variables. Spearheaded by the International Headache Genetics Consortium (IHGC; www.headachegenetics.org/), which brought together headache geneticists and clinicians from around the globe, various large-scale association studies were conducted. Here we will review the main findings of their studies: the DNA variants that were identified, what efforts were made to link these to genes and molecular pathways, and whether any hypotheses emerged that may guide the development of migraine treatments.

## Main text

### Genome-wide association studies in migraine

In the past decade IHGC researchers have conducted several GWAS for migraine (for review see [10, 12]). With increasing samples sizes available for investigation, the number of associated gene variants also increased. Initial sample sizes consisted of a few thousand patients with migraine with aura (in the 2010 GWAS [13]) or migraine without aura (in the 2012 GWAS [14]) that had been recruited in specialised headache clinics and yielded one and six associated SNPs, respectively. A SNP refers to a specific location on the genome with two alleles, indicated by an rs-number (e.g., rs9349379, the Reference SNP cluster ID number for that SNP that is searchable in the SNP database [dbSNP: www.ncbi.nlm.nih.gov/SNP/]). In the 2011 GWAS [15], 5122 women with migraine from the Women’s Genome Health Study were investigated and three associated SNPs were identified, two of which surfaced also in the 2012 GWAS. In more recent efforts, meta-analyses were performed on genotypic data of the previous cohorts that was combined with data of other cohorts to yield much larger groups of migraine patients; i.e., 23,285 cases in the 2013 GWAS [16] and 59,674 cases in the 2016 GWAS) [17], which led to 13 and 44 associated SNPs, respectively. In GWAS, genotypic information of migraine cases is compared with data of ever-increasing numbers (in the latest study 316,078) of control subjects. Notably, it is customary to not screen for (and remove) cases (~ 15% in the case of migraine) from the control sets that typically are from large population-based cohorts. An important message from these GWAS is that migraine-associated SNPs are generally very robust findings due to their stringent statistical methodology and consequently most of them have been replicated in subsequent studies. Secondly, all associated SNPs have a small genetic effect with allelic odds ratio of 1.03–1.28 (for the disease-increasing risk allele) [13–17], which resonates earlier claims that no single genetic factor is sufficient to cause migraine, which is no different for any other disorder studied with GWAS [18].

### The difficult road from associated SNPs to genes and mechanisms

Whereas there is little doubt that the identified variants (indicated by their rs ID number) are genuine findings, robustly linking those variants to genes and pathways is difficult due to the complexity of local genomic effects. Firstly, most attention in literature goes to reporting the index SNP (i.e., the SNP with the lowest *p*-value in a genomic region), but there can be multiple independent association signals at the same locus, called secondary SNPs, which may affect, for example, other regulatory features of the same (or neighbouring) gene. The 2016 GWAS, with its 44 migraine-associated SNPs (associations were with the subtype migraine without aura), implicated 38 distinct genomic loci, of which six contained an independent secondary signal (Table 1).

**Table 1:** Migraine-associated single nucleotide polymorphisms and the molecular pathways they are linked to

Genomic region^a^	Index SNP^b^	Secondary SNP^c^	Gene nearest index SNP	Genes overlapping credible SNPs	Genes prioritised with DEPICT	Pathways identified with g:GOSt tool
1	rs10218452		*PRDM16*	*PRDM16*	*PRDM16*	Vascular function; Metal ion homeostasis
		rs12135062				
2	rs1572668		*LRRIQ3*			
3	rs2078371		*TSPAN2*		*NGF*	
		rs7544256				
4	rs6693567		*ADAMTSL4*	*RPRD2*	*ECM1*	Vascular function
5	rs1925950		*MEF2D*	*MEF2D*		Vascular function
6	rs138556413		*CARF*	*CARF*	*NBEAL1*	
7	rs10166942		*TRPM8*	*TRPM8*		Ion channel activity
		rs566529				
8	rs6791480		*TGFBR2*		*TGFBR2*	Vascular function; Metal ion homeostasis
9	rs13078967		*GPR149*		*ARHGEF26*	Vascular function
10	rs7684253		*SPINK2*		*REST*	Vascular function; Metal ion homeostasis; Ion channel activity
11	rs9349379		*PHACTR1*	*PHACTR1*		Vascular function
12	rs140002913		*NOTCH4*			
13	rs10456100		*KCNK5*	*KCNK5*		Ion channel activity
14	rs67338227		*FHL5*	*FHL5*		Vascular function
		rs4839827				
15	rs28455731		*GJA1*		*GJA1*	Vascular function
16	rs1268083		*HEY2*	*HEY2, NCOA7*	*HEY2*	Vascular function
17	rs186166891		*SUGCT*	*SUGCT*		
18	rs10155855		*DOCK4*			
19	rs6478241		*ASTN2*	*ASTN2*		
20	rs2506142		*NRP1*	*NRP1*	*NRP1*	Vascular function; Metal ion homeostasis
21	rs10786156		*PLCE1*	*PLCE1*	*PLCE1*	Vascular function
		rs75473620				
22	rs12260159		*HPSE2*	*HPSE2*	*HPSE2*	
23	rs2223089		*ARMS2*	*PLEKHA1, HTRA1*	*HTRA1*	Vascular function
24	rs4910165		*MRVI1*	*MRVI1*	*MRVI1*	
25	rs11031122		*MPPED2*	*MPPED2*		
26	rs10895275		*YAP1*	*YAP1*	*YAP1*	Vascular function
27	rs561561		*IGSF9B*	*IGSF9B*		
28	rs1024905		*FGF6*		*FGF6*	Vascular function
29	rs11172113		*LRP1*	*LRP1*	*LRP1*	Vascular function; Metal ion homeostasis
		rs11172055				
30	rs11624776		*ITPK1*			
31	rs77505915		*CFDP1*	*CFDP1, TMEM170A*		
32	rs4081947		*ZCCHC14*		*ZCCHC14*	Vascular function; Metal ion homeostasis
33	rs75213074		*WSCD1*			
34	rs17857135		*RNF213*	*RNF213*		Metal ion homeostasis
35	rs111404218		*JAG1*		*JAG1*	Vascular function; Metal ion homeostasis
36	rs4814864		*SLC24A3*	*SLC24A3*		Ion channel activity
37	rs144017103		*CCM2L*	*CCM2L*	*CCM2L*	Vascular function
38	rs12845494		*MED14*			

^a^Genomic region is an independent genomic region (> 250 kb apart) that harbours at least one migraine risk SNP; ^b^Index SNP is the SNP with the lowest *p*-value at a genomic region. ^c^Secondary SNP is a genome-wide significant SNP that is not in linkage disequilibrium with the index SNP. Associations were identified for the migraine without aura subtype. DEPICT, data-driven expression-prioritized integration for complex traits; g:GOSt tool refers to web-based gene functional profiling software g:Profiler128 (http://biit.cs.ut.ee/gprofiler/) (depicted are only the more prominent pathways vascular function, metal ion homeostasis, ion channel activity pathways) (Compiled and adapted from [17, 33])

Secondly, traditionally the most straightforward approach in interpreting a GWAS signal was to link the index SNP to the *nearest* gene, under evidence that regulatory effects tend to largely act on short distances [19, 20]. The strength of this inference depends on a number of factors, such as the size and gene density of the identified locus; while long-range *trans-*eQTL (‘expression quantitative trait loci’ which explain small fractions of the genetic variance of a gene expression phenotype) effects exist in the genome, the preponderance towards short distance *cis-*eQTLs suggest we can be fairly confident in linking a locus where only a single gene resides within the associated SNPs to hypothesise about function. One way to combat this is to combine the evidence from association-test statistics with linkage disequilibrium information (i.e., prior information on how haplotype patterns [alleles of close by SNPs] behave at each locus) in a Bayesian approach [21], to define what is called a *credible set of SNPs* (i.e., the set of SNPs that with a 99% chance contain the causal SNP at a locus). Using the genomic location of the credible SNPs the most likely gene(s) associated with migraine were identified (Table 1). Since causal variants are often located in intronic or intergenic regions in gene-dense areas, inferring which of the many genes within the credible set is involved based on SNP data alone can be tricky. In practice, this means that all the genes at such loci need to be taken forward to post-hoc analyses, which imposes power challenges to such analyses. Information on the gene’s function and participation in known biological pathways can be used to prioritise causative genes using methods such as DEPICT [22], which prioritises genes if their predicted function is shared with that of genes at other associated loci more often than expected. Together, analysis of credible SNPs and DEPICT analysis identified 37 genes that are likely to be causal (Table 1).

Thirdly, even in cases where only a single gene is implicated, the complexity of gene regulation can provide additional challenges; this was clearly demonstrated by the fact that intronic SNP rs9349379 that had been linked in this way to *PHACTR1* in multiple migraine GWAS (as well as coronary artery disease, cervical artery dissection, fibro-muscular dysplasia, and hypertension) and where the credible set comprises only rs9349379, detailed functional follow-up analyses revealed that this SNP influences the expression of *EDN1* (coding for endothelin-1 (ET-1), 600,000 base pairs [bp] upstream) [23]. ET-1 is a potent vasoconstrictor that acts on smooth muscle cells and has previously been implicated in migraine [24]. Although hypothesis-free methods such as GWAS and sequencing studies are meant to provide the roadmap towards core pathophysiology of a disease, they rely on direct and well-designed ‘wet-lab’ functional follow-ups to nail down the key molecular mechanisms. As the type of assay, whether animal- or cell-based, needed for a functional follow-up very much depends on the actual variant and the gene it affects, it is not possible to give specific directions on how to go about for a particular variant (for recent reviews on technical possibilities see [25, 26]).

### Emerging molecular pathways from GWAS hits?

The most profound hypothesis that emerged from the 2016 IHGC GWAS publication [17] (with only a minute fraction of the genes/loci identified thus far) was the enrichment of genes involved in the vascular system among the identified genetic risk factors for migraine. Briefly, tissue expression enrichment analysis was performed, where the expression of genes (from GTEx data) within 50,000 bp of credible-set SNPs was assessed in 42 different human tissue types. These analyses identified that arterial and gastrointestinal tissues were significantly enriched for expression of migraine-associated genes. Indeed, no less than 15 of the implicated genes are related to vascular function of which four (*MEF2D, YAP1*, *LRP1, JAG1*) were significantly enriched in vascular tissues, as shown by in silico tissue expression enrichment analysis [17].

The 2016 gene expression enrichment results suggested that vascular dysfunction is important in migraine susceptibility and fuelled the long-running debate whether migraine is a disease of vascular dysfunction, or of neuronal dysfunction with vascular changes playing a secondary role. However, the 2016 finding by no means suggests that a neuronal origin of migraine is now excluded, already because at least five genes (*PRDM16, MEF2D, FHL5, ASTN2, LRP1*) (also) have a neuronal function. Another, rather unexpected, hypothesis that emerges is that metal ion homeostasis might contribute to migraine susceptibility, as 11 genes (*PRDM16, TGFBR2, REST, FHL5, NRP1, MMPED2, LRP1, ZCCHC14, RNF213, JAG1, SLC24A3*) with such function are among the 37 genes. Of note, ion channel activity (*TRPM8, REST, KCNK5, SLC24A3*), which emerged from genetic studies in monogenic FHM, and pain signalling (*TRPM8*) were much less prominent signals [27].

A more recent [28] tissue enrichment analysis of the 2016 IHGC GWAS summary statistics ― utilising two gene expression datasets (GTEx and ‘Franke lab’) and chromatin data (highlighting active regulatory regions) from the Roadmap Epigenomics and ENCODE (EN-TEx) projects ― reported enrichment of both vascular and neurological enrichment. More specifically, cardiovascular enrichments were found for migraine without aura with gene expression data, and for migraine without aura and ‘all’ migraine with EN-TEx data. Whereas, analysis using Roadmap data found the strongest enrichment for migraine (all subtypes) was neurological (neurospheres and fetal brain, neither of which were present in GTEx and EN-TEx). These results highlight the importance utilising multiple tissues, cell types and regulatory measures in such enrichment analyses aimed at interpreting GWAS risk loci.

It is useful to keep in mind that a GWAS SNP only ‘tags’ the disease locus, implying that the identified SNP is only correlated — because of linkage disequilibrium — with the disease-causing variant, which is not ‘the end of the road’ as far as understanding the functional consequences. Efforts at combining information across phenotypes either directly at the summary statistic phase [29] or by comparative analysis of correlated phenotypes [30] as well as increasing the size of the migraine GWAS itself (leading to more implicated loci) will yield improvements on the locus side of the analysis; concurrently, considerable efforts are being focused on improving the quality of the next layer of information, which links SNPs to function, through various -omics studies assaying the genome in general [26, 31], and the improvement of these resources and better methodology will increase the statistical power on the post-hoc side of the analysis. However, it is crucial to realise that rapid progress can also be made in migraine specifically by targeted follow-ups (such as for the rs9349379/EDN1/ET-1 study) [23], given that we now have a set of well-characterised loci waiting for such detailed characterisation. For example, several of the mechanisms implied by the two latest GWAS (such as regulation of vascular tone, ion homeostasis) may present directly testable hypotheses.

### What lies ahead?

GWAS in migraine have been fruitful in the sense that they yielded several dozens of robustly identified loci in the genome that harbour genetic risk factors. Despite clear challenges how to link associated SNPs to actual genes and pathways, the likelihood that the correct genes are identified is increased by bioinformatics tools. Emerging hypotheses suggest that vascular function and metal ion homeostasis are among the pathways involved in migraine pathophysiology. Other pathways such as neuronal function and ion channel activity are less prominent among the genes identified thus far. Current initiatives of IHGC to conduct even larger GWAS (close to 100 K cases) appear to identify many more risk loci (> 100) [32] that may support current hypotheses and likely generate new ones. Over time, the genetic landscape of migraine will be more complete so one may predict migraine risk using approaches like polygenic risk scores, which is not yet sufficiently accurate [33–35]. One major challenge will be to elucidate the functional consequences of the associated SNPs and identify how they may affect migraine risk at the individual level. Efforts to functionally characterise GWAS signals, for other diseases than migraine, have been considering high-throughput cell-based (e.g., induced pluripotent stem cells [iPSCs]) and animal models (e.g., Drosophila) [26]. Considerable amount of research is needed before migraine GWAS findings will show diagnostic or prognostic value and lead to the development of (personalised) treatment options.

## Abbreviations

CADASIL: Cerebral autosomal dominant arteriopathy with subcortical infarcts and leukoencephalopathy
CSD: Cortical spreading depolarisation
DEPICT: Data-driven expression-prioritized integration for complex traits
FASPS: Familial advanced sleep phase syndrome
FHM: Familial hemiplegic migraine
GWAS: Genome-wide association study
IHGC: International Headache Genetics Consortium
SNP: Single nucleotide polymorphism
